# Notes and News

**Published:** 1887-01-01

**Authors:** 


### Notes and News.

Collected and Communicated.

It has been decided by the Governors of the Princess Alice Memorial Hospital at Eastbourne to enlarge the present building, which is often crowded.

The Hospital for Consumption, Brompton, of which Mr. Henry Dobbin is the experienced and able secretary, appeals urgently for help at this season. It is the oldest, and probably the best hospital of the kind in this country, and no one having money to give, can possibly do better than forward a cheque to Mr. Dobbin without delay.

The lease of the present building of the British Home for Incurables at Clapham Rise, will, we are informed, soon fall in, and a new home will have to be found.

The Worshipful Company of Saddlers have sent twenty guineas, and the Benchers of the Honourable Society of Gray's Inn ten guineas, to the fund now being raised for the Great Northern Central Hospital new building.

The Chester Infirmary has received a cheque for £500 from the Duke of Westminster, being the proceeds of the shilling entrance fees paid by visitors who have inspected Eaton Hall during the past year.

The Committee of the Royal Westminster Ophthalmic Hospital are anxious, if possible, to re-open the two wards (one for males, and the other for females), which have been so long closed at their institution, in order to meet the pressing demands of the poor. About £2,000 is needed to justify them in taking this step. The hospital has thirteen wards, capable of receiving about fifty cases at a time.

The Committee of the Royal Hospital for Children and Women are striving at the present time to raise a thousand pounds to clear off some pressing liabilities. The hospital, which is situated in the Westminster Bridge Road, in the midst of a very poor and a very populous neighbourhood, was established in 1816, and ranks as the oldest institution of its kind in London. It has now six wards, containing 53 beds for in-patients, who average over 400 a year, while the out-patients number between six and seven thousand.

The great success of last year's ball in aid of the Chelsea Hospital for Women has induced the Committee to repeat the experiment. This year the Fancy Dress Ball and Children's Christmas Party is to be held in that superb *suite* of apartments known as the Whitehall Rooms, at the Hotel Métropole. The floor of this grand ball-room has been specially laid for dancing, and is in perfect order. The principal dining-room and the drawing-room will be used for the supper and retiring rooms. The ball, which has been fixed for January 13th, promises to be even a greater success than that of last year.

Children suffering from bodily deformities through no fault of their own, cannot fail to excite our sympathy. To see some of the poor little sufferers, whose friends are endeavouring to obtain for them admission into the City Orthopædic Hospital, is a sight painful in the extreme. But it is yet more sad when one learns that many of these children, suffering in some cases from most agonising deformities of their little frames, are unable to obtain admission from the simple fact that the funds of the hospital will not allow it. At the present time, there are nearly three hundred children waiting to get in, some of whom are in cruel torture from hip disease and distortions of the limbs.

The grave pecuniary condition of Guy's Hospital has already loosened the purse-strings of many of the wealthy supporters of this splendid institution. A hundred thousand pounds is a big sum to raise, but with the judicious selection of representative men who will give time to the work, and their election as governors, the necessary funds ought to be forthcoming. Already some of the wards have been closed; a further curtailment of the resources of this institution would be little short of a public calamity to the poor inhabitants of the Borough and its neighbourhood. "Sweet are the uses of adversity." The Trustees of Guy's should take this trite truth to heart, and ask themselves how it is that the landed wealth of Guy's has declined in value from £41,000 per annum, at which it stood ten years ago, to £26,000, the total available income to-day? But, in the evil hour that has come, it is little use to discuss what should have been. Two things may be done, however, and success secured. First, every member of the governing body should be a working member; and secondly, steps should be taken to give the public who are now asked to subscribe some voice and interest in the management. No doubt some of our readers will say to themselves, "Is that really all?" Yes, it really is all that is necessary to ensure that the public will place Guy's once and for all upon a sound financial basis.

It is not, we believe, so generally known as it should be, that Guy's, St. Thomas's and St. Bartholomew's, being endowed charities, receive nothing whatever from the Hospital Sunday Fund. No doubt one reason is that the plan of distribution provides that grants to participating charities shall be based upon the voluntary income. So that the sum which the endowed hospitals would be entitled to receive from the Hospital Sunday Fund, if they participated, would be relatively inappreciable and small.

On three recent evenings the Students' Club of the Charing Cross Hospital gave a series of entertainments to the patients in the hospital. At the last of the series a number of friends attended, in addition to the patients. The programme comprised songs, recitations, and music. The orchestra was under the direction of Dr. Mott, who acted as stage-manager in the two plays performed, viz., "A Husband to Order" and "Nursery Chickweed." The whole of the performance was undertaken by the students and staff, with the assistance of some ladies, who volunteered their kindly help. We understand this is the eighth annual entertainment given by this club, and we trust that they may go on and prosper in their good work of cheering the sick.

Mr. G. Lawson, F.R.C.S., Surgeon Oculist to the Queen, has consented to act as consulting ophthalmic surgeon to the Hospital for Epilepsy and Paralysis, Regent's Park, *vice* Mr. White Cooper, deceased. Mr. George Lawson is one of the most eminent of surgeons as he is one of the kindest of men, and the authorities of the Hospital for Epilepsy and Paralysis are to be congratulated on their good fortune in securing so valuable an addition to their staff.

A young English lady, who is studying music in Berlin, sends us an earnest appeal on behalf of a new "Home for English and American Girls," which has just been opened there, under the patronage of H.R.H. the Crown Princess of Germany. The want of a safe and pleasant home, where the cost of living would be moderate, has long been felt by the relatives and friends of English-speaking girls who go over to Berlin to complete their studies. Nothing could be more undesirable than the present system of allowing the young lady students to live in pensions and lodging-houses, subject to no control except their own sweet will. That the Crown Princess takes a lively interest in the new scheme, and has herself appointed the Lady-Principal of it (Fräulein Selka), is a guarantee of its respectability in this country; for members of the English Royal Family deservedly possess the full confidence of their fellow-countrymen in their good sense and discretion. The appeal now made is for sufficient funds to set the concern in thorough going order, after which it will be self-supporting. Subsequent contributions will not be discouraged, however; as, apart from the purpose of comfortably housing girl-students who can well afford to pay for the privilege, it is especially intended that this home should be a place where governesses looking for employment, or requiring a holiday-retreat, may be properly cared for, without too great a strain being placed upon their moderate resources. Indeed, it is suggested that a payment of two shillings a day should cover every expense incidental to board and lodging in such cases.

Our authority for the above information is indisputable, and we recommend the Home to the kindly consideration of our charitable readers. If subscriptions are sent to the Editor of The Hospital, they will be duly forwarded; but it might be better to send them direct to "Frl. Selka, *per add.* Frl. Strahl, Berlin, Schill Strasse, 4."

A conference of delegates was held at Birmingham on the 11th December, to consider a proposal for the establishment of a Convalescent Home for members of the Ancient Order of Foresters in the Midlands. Eighty-four courts, comprising nearly thirty thousand members, were represented. The district committee suggested that an eight-roomed house should be taken for the purposes of the Home, and estimated the total cost at £500 per annum, which could be covered by levying one penny a quarter on each member in the Midlands. The scheme was approved, and a committee appointed to consider and deal with the necessary details.

Bristol proposes to celebrate the Jubilee by the establishment of a "Queen Victoria's Maternity Hospital," its inhabitants not at present possessing any institution of the kind. We believe the chief medical authorities have often proclaimed their sense of the crying need for some such provision, and we hope the project will be carried through successfully. It is stated that Her Majesty will be asked to lay the foundation stone herself.

Mr. D. P. Loe has promised to bear the entire cost of furnishing the new wing which has recently been added to the Bromley Cottage Hospital, provided the debt of £750, still owing for the cost of building it, is wiped off by the 31st January, 1887. A donation of £50 towards this end has been given by Mr. John Scott, of Albyfield, Bickley, and the committee earnestly hope that the charitable may yet enable them to accept Mr. Loe's generous offer.

Colonel Milman has received the munificent donation of £500 towards the building fund of the Great Northern Central Hospital, from H. A. Tufnell, Esq., lord of the adjoining manor, who has also consented to be President of the Camden District Committee.

Mr. Thomas H. Cox, of Dundee, has made the magnificent gift of £12,000 to the Dundee University, for the foundation of a Chair of Anatomy in the medical school, which it is proposed to form in connection with the college.

A vigorous movement is being made in Hull to rebuild, on a much enlarged and improved scale, the Children's Hospital, now housed in Story Street. A capital site has been obtained in Park Street, at a very moderate cost, which will be sufficient for a hospital with fifty cots, and an isolated ward for infectious cases. Altogether, about £5,000 is required. For the past two or three years the present little hospital has been quite inadequate for the wants of a town of such rapid growth as Hull, despite the accommodation for children which exists at the Hull Infirmary. The "third seaport" of the United Kingdom could, we should think, find no more fitting memorial of Her Majesty's Jubilee than such a hospital for the sick children of the poor, and we hope that the inhabitants of the town will give the movement their undivided support. The manhood of the coming generation depends, to a very large extent, upon the health of present childhood. This fact the people of Hull would do well to bear in mind.

The Annual Report, just issued, of the Chelsea Hospital for Women, is, on the whole, one of an encouraging character. This useful institution, which is situated on the Fulham Road, just by the Queen's Elm, was founded in 1871, for the treatment of poor women and distressed gentlewomen suffering from diseases peculiar to their sex. The hospital has a complement of 63

beds, but for nearly two years the wards on the Albert Edward floor, numbering 23 beds, had to be entirely closed. During the past year, however, the managers have thrown these open; the in-patients during the twelve months numbering 457, as against 339 in the preceding year, and 265 the year before. For some reason, difficult to assign, the out-patient department during the year has not been quite so numerously attended.

---

The North-Eastern Hospital for Children is just now in urgent need of help, to meet the Christmas accounts. During the past year there has been an exceptional call upon the resources of this institution, which has raised out-goings much above the in-comings. The hospital, which was established in 1867, entirely for the relief of the sick children of the poor, is situated in the Hackney Road, in the midst of the densely populated districts of Shoreditch, Bethnal Green, Hackney, Haggerston, and Hoxton. During the past twelve months the attendance at the out-patient department has averaged about a thousand per week.

---

The Swansea Eye Hospital, which serves the needs not only of Glamorganshire, but of the adjacent counties as well, is much in need of better and more extensive premises to meet the growing demands made upon it. An excellent site has been offered by Mr. J. C. Richardson, at an exceptionally low figure, and it is hoped before long to commence the new building. The Swansea Eye Hospital was founded in 1879, and patients now seek it from all parts of South Wales.

---

It seems at first sight a curious thing that an institution which was originally built to provide accommodation for cases of fever or other infectious diseases, should now think of adding a special ward for the reception of such cases. This, however, is now proposed with respect to Nairn Hospital, which, from a paucity of fever-patients, has gradually become a general hospital, in which the largest number of patients are persons suffering from accidents of various kinds. A representative meeting was recently held at Nairn to consider the propriety of adding a special fever and cholera ward; and, though some doubt of its necessity is felt and expressed in the district, we understand the matter is likely to be carried through very shortly.

---

At the December monthly meeting of the Board of the Blackburn Infirmary, it was stated that the executors of the late Mr. Henry Hargreaves had paid in the legacy of £5,000 left to the institution by that gentleman; and that donations of £50 and £100 had been received from the East Lancashire Football Charity Cup Committee, and from Mr. Richard Edmondson of Witton, respectively.

---

We are sorry to see, from a statement made by Mr. C. T. Murdoch, M.P., the Chairman of Committee of the Great Northern Central Hospital, that the building fund for the new institution now in course of erection, is making but slow progress. We have already pointed out in these columns the urgent need there is for an extension of hospital accommodation in North London. One bed for every 25,000 inhabitants is surely an inadequate provision, and that, too, in a district so largely populated by the working classes. The present hospital has 32 beds, while the new hospital would allow space for 150. The Duke of Westminster, the Baroness Burdett-Coutts, and other philanthropists are warmly supporting the effort which is being made to raise the £45,000 required.

---

Miss Frances Egston, of the Boston Cottage Hospital, sends us the following amusing story. It is to be hoped that it is not typical of the knowledge of the culinary art generally possessed by the wives of our agricultural labourers:—"Now, Smith, you must be careful about your diet," said Dr. —— to one of his patients, an agricultural labourer, one morning. "Get your wife to make you some beef-tea. I suppose you know how?"—turning to the woman as he spoke. "Really, Doctor, of course I do," answered Mrs. Smith, in a highly aggrieved tone. Next day Dr. —— inquired how Smith liked the beef-tea. "Well," said Smith, "the first cup I had was rare and good; but when my wife put the milk and sugar in, it was rare and nasty."

---

It seems now to be a mistake to suppose that doctors are over-paid servants, except perhaps the fortunate few who enjoy the patronage of the upper ten. Sir Spencer Wells, the other day, said he knew a medical practitioner who had been able to get only £100 out of £1,400 that was owing to him. Cases of this kind—and some even far worse—are by no means singular. The competition among doctors is quite as keen as among members of other professions: nor do we believe that things will improve so long as persons, who can and ought to pay for medical advice, are able to obtain all that they require at some of our hospitals and free dispensaries, and yet keep their money in their pockets.

---

Mr. W. Bristow, the Hon. Sec., has issued a "Christmas Appeal" for funds in aid of the Miller Memorial Hospital, Greenwich. During the past year 153 patients have been admitted, 331 accidents have received attention, and over 10,000 out-patients have obtained medical treatment. The erection of another wing, with a circular ward for twenty beds, would greatly add to the accommodation; but at present the committee do not feel justified in incurring this responsibility, as the support the hospital has hitherto received does not keep pace with the necessary expenditure. We hope that aid for this deserving institution will not be sought in vain.

---

Aberdeen has decided to renovate and enlarge the Royal Infirmary, by way of commemorating the fiftieth year of Her Majesty's reign. It is estimated that the work will cost between £20,000 and £30,000. The Infirmary, to which the Queen is herself an annual subscriber, is much in need of extension and improvement in many directions, and the people of Aberdeen have, we think, acted wisely in adopting the "happy inspiration" of Lord Provost Henderson, who has given £1,000. A liberal donor has also sent £550.

The managers of the East London Hospital at Shadwell are very anxious to improve their out-patient accommodation, which for a long time past has proved very inadequate. Plans have been prepared, but the want of funds "bars the way." In the thickly populated district of Shadwell, the East London Hospital is carrying on a most excellent work upon lines of the strictest economy, and it is therefore to be hoped that generous donors will assist the managers to build their out-patients' hall.

---

Ancoats, which has been aptly termed the "workshop of Manchester," is struggling hard to keep its little hospital on its feet; but, somehow or other, the wealthy residents of the great cotton town appear to forget its existence. Everybody above the wage-earning class has long ago bidden adieu to poor Ancoats, and hence the hard times which are pressing on the hospital. This institution now contains thirty-five beds, and much need is felt for a female medical ward. The Ancoats Hospital has a strong claim on the benevolence of the merchant princes of Manchester.

---

A special meeting was recently held of the governors and subscribers of the Newark Hospital and Dispensary, an institution which, it seems, is spending £1,500 a year, balanced only by an income of £1,000. To sell out stock in order to meet current expenses should be a very exceptional course in any hospital which does not want to come to grief. A very long discussion ended in a decision to make a desperate effort, by a house-to-house canvass, to make up the annual deficiency. This effort might, we think, have been seconded by retrenchment in two or three directions, without in the least interfering with the efficacy of the hospital.

---

Children can do much on behalf of hospitals, if only their little efforts are controlled and organised. The Children's Ministering Angels' League, which has branches in different parts of London, is an example of this. The proprietors of two or three children's papers have, with great success, helped the hospitals with sundry useful little gifts, the handiwork of their juvenile readers. The editor of *Little Folks*, which has for several years past thus assisted the children's hospitals, has just distributed a huge collection of painting books, dolls, scrap-albums, needlework, etc., which his little readers have sent him to cheer at Christmas the lot of their less fortunate little brothers and sisters.

---

Mr. H. Allison, Chairman of the Executive Committee, presided at a recent special meeting of the supporters of the Hull Children's Hospital, held for the purpose of considering the purchase of a suitable plot of ground for a new hospital. The price asked for the proposed site was £1,358, and the purchase had already been provisionally completed by the Committee, who suggested the erection of a hospital for fifty cots, with an isolated building for infectious cases, and an out-patient department separate from the main building. The total cost was estimated at £5,000. The action of the Committee was confirmed by the general meeting, and it is hoped that the necessary amount will be speedily collected, so that the building may be completed within the current year.

---

The new wing recently added to the Ealing Cottage Hospital provides accommodation for five extra beds, bringing up the total to fourteen. The hospital is still unfortunately hampered by a debt of £750, which the Committee are striving to clear off.

---

The great metropolis is a city of "many nations." For many years past the French and the Germans resident among us have each possessed an institution devoted specially to their wants. Not very long since the Italians (of whom London musters a goodly number) established in Queen Square, Bloomsbury, an Ospedale Italiano. Though primarily intended for sick Italians, the young hospital, with true humanitarianism, has bestowed its medical and surgical skill upon all comers almost without discrimination. It is hardly surprising, therefore, that the little hospital in Queen Square is unequal to the rapidly increasing demands which are being made upon it. The founders of the Italian Hospital Fund have accordingly under consideration a scheme for the erection of a large and more suitable building.

---

There really can be no two opinions as to the propriety of the course taken by the gentlemen who are organising the movement to present Sir Andrew Clark with some slight testimonial to his distinguished services in the cause of medical education. Though he has not, perhaps, fanned his name from pole to pole by any signal discovery or invention, his services have been none the less valuable. For thirty-two years Sir Andrew Clark has been associated with the London Hospital, and it was only a few weeks since that he resigned the senior physicianship. Though Sir Andrew is now on the list of "consulting physicians," he still goes down to his old institution. The proposed testimonial has already met with warm approval, both among the "old students" of the hospital and others. It is proposed that the presentation shall take the form of a portrait in oil of Sir Andrew, executed by a distinguished artist, and, if funds will allow, of a replica, to be hung in the Library of the new Medical College at the London.

---

Mr. Forster Green is a man of whom Belfast may well be proud. Twelve months ago the Thronemount Consumption Hospital was opened with an endowment—to which Mr. Green was the largest contributor—sufficient to maintain six beds. The Committee, relying on the generosity of the public, ventured to provide eight, and these have been constantly full. At the annual meeting, held the other day, Mr. Green again came forward with a princely donation of £1,000 to build a new wing, conditionally on the public securing its endowment. The need for further provision for the poor suffering from this insidious malady will be apparent when we state that at present scarcely a tenth of the deserving poor who seek admission can be received. Already nearly a thousand pounds has been subscribed to the endowment fund.

